# Association between dialysate sodium concentration and interdialytic weight gain in patients undergoing twice weekly haemodialysis

**DOI:** 10.1186/s12882-021-02401-2

**Published:** 2021-06-17

**Authors:** Soraiya Manji, Jasmit Shah, Ahmed Twahir, Ahmed Sokwala

**Affiliations:** Department of Medicine, The Aga University, Nairobi, Kenya

**Keywords:** Dialysate sodium concentration, Interdialytic weight gain, Blood pressure

## Abstract

**Background:**

Chronic kidney disease is highly prevalent across the globe with more than 2 million people worldwide requiring renal replacement therapy. Interdialytic weight gain is the change in body weight between two sessions of haemodialysis. Higher interdialytic weight gain has been associated with an increase in mortality and adverse cardiovascular outcomes. It has long been questioned whether using a lower dialysate sodium concentration during dialysis would reduce the interdialytic weight gain and hence prevent these adverse outcomes.

**Methods:**

This study was a single blinded cross-over study of patients undergoing twice weekly haemodialysis at the Aga Khan University Hospital, Nairobi and Parklands Kidney Centre. It was conducted over a twelve-week period and patients were divided into two groups: dialysate sodium concentration of 137 meq/l and 140 meq/l. These groups switched over after a six-week period without a washout period. Univariate analysis was conducted using Fisher’s exact test for categorical data and Mann Whitney test for continuous data.

**Results:**

Forty-one patients were included in the analysis. The mean age was 61.37 years, and 73% were males. The mean duration for dialysis was 2.53 years. The interdialytic weight gain was not significantly different between the two groups (2.14 for the 137 meq/l group and 2.35 for the 140 meq/l group, *p* = 0.970). Mean blood pressures were as follows: pre-dialysis: DNa 137 meq/l: systolic 152.14 ± 19.99, diastolic 78.99 ± 12.20, DNa 140 meq/l: systolic 156.95 ± 26.45, diastolic 79.75 ± 11.25 (*p* = 0.379, 0.629 respectively). Post-dialysis: DNa 137 meq/l: systolic 147.29 ± 22.22, diastolic 77.85 ± 12.82 DNa 140 meq/l: systolic 151.48 ± 25.65, diastolic 79.66 ± 15.78 (*p* = 0.569, 0.621 respectively).

**Conclusion:**

There was no significant difference in the interdialytic weight gain as well as pre dialysis and post dialysis systolic and diastolic blood pressures between the two groups. Therefore, using a lower dialysate sodium concentration does not appear useful in altering the interdialytic weight gain or blood pressure although further studies are warranted with a larger sample size, taking into account residual renal function and longer duration for impact on blood pressures.

## Background

Chronic kidney disease (CKD) is the presence of kidney damage or decreased kidney function for three or more months**,** irrespective of the cause. Globally CKD has a prevalence of 13.4%, with 10.6% being in stage 3–5 of the Kidney Disease Improving Global Outcomes (KDIGO) classification of CKD between the year 2000 to 2014 [1]. The mortality rate for patients with CKD globally is also high, particularly for those on renal replacement therapy [2]. More than 2 million people globally are requiring renal replacement therapy. However, there is less renal replacement therapy in the developing world due to lack of access and affordability issue [3]. This has resulted in patients with End Stage Renal Disease (ESRD) having less than the recommended number of sessions of dialysis. The current recommendations as per the Kidney Disease Outcomes Quality Initiative (KDOQI) guidelines for haemodialysis adequacy are geared towards more frequent and shorter duration of dialysis [4]. Unfortunately, this is far from the case in sub-Saharan Africa (SSA). In a study done at Kenyatta National Hospital in Kenya, 98.15% of patients underwent haemodialysis less than three times a week [5]. The health insurance scheme in Kenya (National Health Insurance Fund - NHIF) covers two sessions of dialysis per week, and those getting three or more sessions per week have to cover the cost of dialysis themselves, which poses a great challenge as far as moving towards reduced morbidity and mortality from ESRD [6].

Usually, the operator of the dialysis machine (nurse) must set the dialysate concentration for the dialysis session. During the process of dialysis, sodium is lost from the blood by ultrafiltration into the dialysate fluid. According to Flythe et al., the dialysate sodium concentration therefore has to be lower than the serum sodium so as to allow diffusion to occur and the serum sodium to be lowered (dialysate sodium at least 2 meq/l lower than serum sodium). Using a higher dialysate sodium concentration results in ‘sodium-loading’ and thus activates the centre of thirst in the hypothalamus which makes the patient drink more water with subsequent weight gain and volume expansion. There is also an increase in sympathetic tone and release of vasopressin which may result in increased blood pressure and cardiovascular sequelae [7]. Moret et al. had a different view on this, focusing on ioninc mass balance, where diffusive ion influx occurred when the dialysate sodium was approximately 5 meq/l greater than the serum sodium [7].

Interdialytic weight gain is ‘the change in body weight between two sessions of haemodialysis [8]. With time, the set dialysate sodium concentration has gradually evolved from 126.5 meq/l in the 1940s, to around 140 meq/l in the 1990s, but there is no consensus on the optimal dialysate sodium concentration [9]. There has been conflicting evidence, but many studies suggest that with a higher dialysate sodium there is a higher interdialytic weight gain and higher blood pressures. There has also been a trend towards increased mortality, heart failure and major cardiac events (nonfatal myocardial infarction, nonfatal ischemic stroke, or cardiovascular death) as well as hospitalisations in patients with a higher interdialytic weight gain [10, 11].

Therefore, the aim of this study was to determine if dialysate sodium concentration has any bearing on interdialytic weight gain and blood pressure control. Most studies that have been done are observational with very few clinical trials. Furthermore, in most centres where these studies were done, patients were undergoing thrice weekly dialysis. In our set-up, majority of our patients undergo twice weekly dialysis, which would predispose them to a higher interdialytic weight gain due to a longer duration between dialysis sessions.

The primary objective was to determine the association between the dialysate sodium concentration and interdialytic weight gain in patients undergoing haemodialysis twice weekly. The secondary objective was to determine the relationship between dialysate sodium concentration and blood pressure in patients undergoing haemodialysis twice weekly.

## Methods

### Study setting

The study setting was the Aga Khan University Hospital dialysis unit and Parklands Kidney Centre. The Aga Khan University Hospital is a private, not-for-profit teaching hospital and the dialysis unit at the hospital has 9 dialysis machines, with approximately 18 patients undergoing dialysis every day. There are approximately 48 patients in total on dialysis at the unit, out of which 30 are on twice-weekly dialysis. Parklands Kidney Centre is an outpatient dialysis unit that has 19 dialysis machines and approximately 90 patients in total, out of which approximately 45 are on twice-weekly dialysis.

### Study design and subjects

The study was a randomized single blind crossover study design. Each group was dialysed using a dialysate sodium of 140 and 137 meq/l at different time periods. The study was conducted at the Aga Khan University Hospital dialysis unit and Parklands Kidney Centre dialysis unit in Nairobi, Kenya on patients undergoing twice weekly dialysis over a period of 12 weeks.

The inclusion criteria was as follows: age greater than 18 years, patients undergoing dialysis twice a week, patients who consented to be a part of the study. The exclusion criteria was as follows: patients with blood pressures less than 100/60, hospital in-patients. Withdrawal criteria was as follows: patients who develop intradialytic hypotension (reduction in systolic blood pressure of 20 mmHg or a reduction in MAP of 10 mmHg accompanied by symptoms such as muscle cramps, abdominal discomfort, dizziness, nausea, vomiting, yawning, sighing, restlessness, or anxiety) [12, 13].

**Fig. 1:**
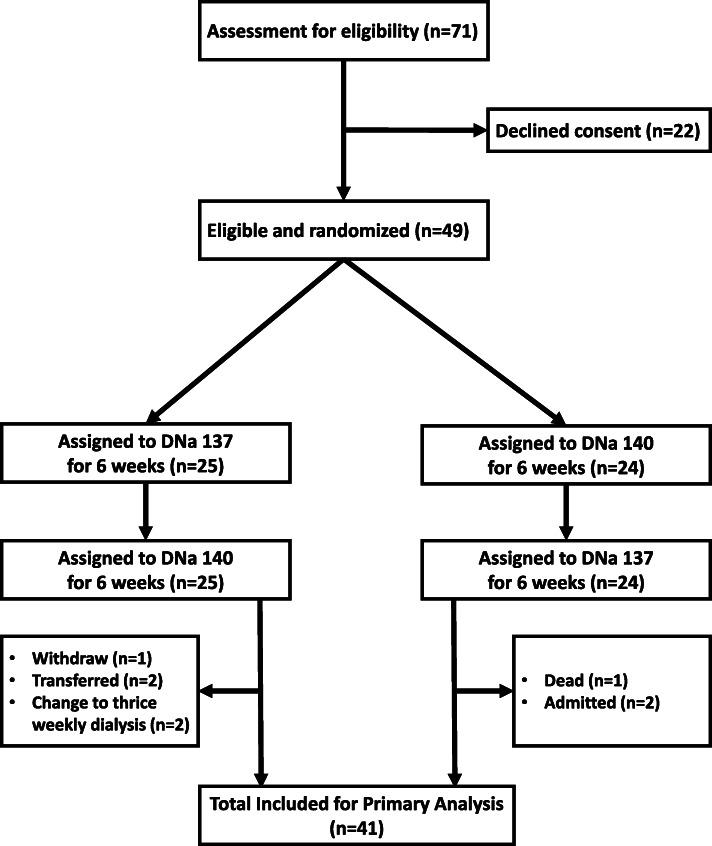
Participant flowchart

Figure 1 shows the study flow. The first group of patients were dialysed using a DNa of 140 meq/l for 6 weeks, followed by 137 meq/l for the remaining 6 weeks. The second group were dialysed using a DNa of 137 meq/l for 6 weeks, followed by 140 meq/l for the remaining 6 weeks. A total of 41 patients were included in the analysis.

### Statistical analysis

Categorical data were presented as frequencies and percentages whereas continuous data were presented as means and standard deviations. Univariate analysis was conducted using Fisher’s exact test for categorical data and Mann Whitney test for continuous data. The comparison between IDWG and mean systolic and diastolic blood pressure with each sodium group chosen for this study was conducted using the mean IDWG and mean systolic/diastolic blood pressure using Mann Whitney test.

## Results

### Demographic and clinical characteristics

The mean age of participants was 61.39 years (SD = 13.82) with a predominantly male population (73%). The most common cause of CKD in these patients was hypertension, followed by diabetes mellitus. Most patients were oliguric. Fifty-nine percent of patients had a fistula for dialysis access, whereas the remainder had internal jugular permanent dialysis catheters. Only 45% of patients were on diuretics. The mean number of years of the patients on dialysis was 2.53. Table 1 shows the demographic and clinical characteristics.

**Table 1:** Demographic and clinical characteristics of study participants

Age (years)	61.39 ± 13.82
Gender	
Female	11 (27%)
Male	30 (73%)
Cause of ESRD (n (%))	
Diabetes mellitus	24 (59%)
Hypertension	33 (80%)
HIV	3 (7%)
Glomerulonephritis	3 (7%)
Multiple myeloma	1 (2%)
Contrast induced nephropathy	1 (2%)
Obstructive uropathy	1 (2%)
Non-steroidal anti inflammatory drugs	2 (5%)
Urine output	
Anuric (< 100 ml/day)	5 (13%)
Non-oliguric (> 400 ml/day)	12 (31%)
Oliguric (100–400 ml/day)	22 (56%)
Dialysis access	
Fistula	24 (59%)
Permanent catheter	17 (41%)
Diuretic use	
No	21 (55%)
Yes	17 (45%)
No. of years on dialysis	2.53 ± 1.96

### Interdialytic weight gain

**Fig. 2:**
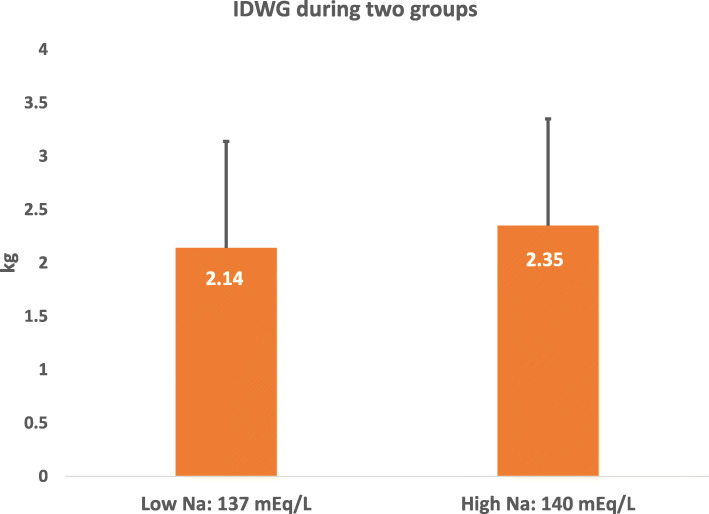
Interdialytic weight gain between DNa 137 meq/l and DNa 140 meq/l

The primary outcome was the interdialytic weight gain between the two groups of dialysate sodium. The average interdialytic weight gain was 2.14 kg in the low dialysate sodium group (DNa 137 meq) and 2.35 in the high dialysate sodium group (DNa 140 meq), with a *p* value of 0.970, as seen in Table 2 and Fig. 2.

**Table 2:** Interdialytic weight gain between DNa 137 meq/l and DNa 140 meq/l

	Low DNa (dialysate Na: 137)	High DNa (dialysate Na: 140)	*P* Value
Previous post dialysis weight (kg)	72.27 ± 16.77	72.24 ± 16.69	0.948
Pre dialysis weight (kg)	75.20 ± 17.23	74.37 ± 17.02	0.856
IDWG (kg)	2.14 ± 1.10	2.35 ± 1.38	0.970

### Blood pressure

The mean pre-dialysis systolic blood pressure was152.14 151.54 in the low DNa group and 156.95156.05 in the high DNa group, while the mean pre-dialysis diastolic blood pressure was 78.99 in the low DNa group and 79.8580.81 in the high DNa group. The average post-dialysis systolic blood pressure was 147.296 in the low DNa group and 151.48 in the high DNa group, while the average post-dialysis diastolic blood pressure was 77.85 in the low DNa group and 79.6680 in the high DNa group. None of these differences were statistically significant between both groups as shown in Table 3.

**Table 3:** Baseline blood pressure, pre-dialysis and post-dialysis systolic and diastolic blood pressures between the two groups

	Low DNa (dialysate Na: 137)	High DNa (dialysate Na: 140)	*P* Value
Baseline SBP (mmHg)	156.24 ± 25.18	156.51 ± 23.43	0.957
Baseline DBP (mm/Hg)	78.54 ± 14.65	77.7 ± 12.88	0.940
Pre-dialysis SBP (mmHg)	152.14 ± 19.99	156.95 ± 26.45	0.379
Pre-dialysis DBP (mmHg)	78.99 ± 12.20	79.75 ± 11.25	0.629
Post-dialysis SBP (mmHg)	147.29 ± 22.22	151.48 ± 25.65	0.569
Post-dialysis DBP (mmHg)	77.85 ± 12.82	79.66 ± 15.78	0.621

Patients with hypertension were subanalysed to determine if there was a difference in weight gain in this subgroup and there was no significant difference.

### Adverse effects

Of the 41 patients, two patients experienced hypotensive episodes. The first patient had asymptomatic hypotensive episodes on their 8th and 12th sessions of dialysis at dialysate sodium concentration of 140 meq/l. The second patient developed an asymptomatic hypotensive episode in the 4th session of dialysis at dialysate sodium concentration of 137 meq/l and a hypotensive episode on the 2nd session of dialysis at dialysate sodium concentration 140 meq/l. The latter required disconnection of the dialysis machine 30 min prior to completion. None of these episodes required saline infusion.

## Discussion

This study has established that there is no association between dialysate sodium concentration and interdialytic weight gain. Furthermore, there was no association between dialysate sodium concentration and blood pressure. This was similar to what was found by Beduschi et al. when comparing DNas of 135 and 138 meq/l where there was no significant difference in the interdialytic weight gain or the blood pressure [11]. This was also the case in the study by Thein et al. where there was no significant difference in the interdialytic weight gain between dialysate sodiums of 141 and 138 meq/l, however there was a significant reduction in blood pressure with the lower dialysate sodium used [13]. As much as there was no statistical significance in the interdialytic weight gain and blood pressure between the two groups, there could still be clinical significance as both parameters were lower in the lower dialysate sodium group.

It is important to note that there was no exclusion of patients with residual renal function in this study, yet residual renal function (defined as urine output of greater than 200 ml/day) theoretically has a physiologic role in sodium balance [14]. Ipema et al. established that patients with residual diuresis had significantly lower interdialytic weight gain [15, 16]. However in terms of outcomes, Hecking et al. noted no difference in mortality in patients with or without residual renal function in spite of the dialysate sodium concentrations used [10]. In the present study, there was no difference in the outcomes in patients with or without residual renal function.

It has also been established in some studies such as that by Titze et al. that large amounts of sodium can be accumulated without water retention by the sodium ions binding to extracellular matrix components such as glycosaminoglycans [17]. In addition, there are other sodium reservoirs in the body such as the bone, skin cartilage and connective tissue and as a result, lowering the dialysate sodium concentration could have caused loss of sodium without loss of water and had no impact on the interdialytic weight gain and blood pressure [11].

There is also theory that every individual has their own individual osmolar setpoint based on parameters such as dietary salt intake, urinary sodium excretion, tissue sodium stores as well as physiologic response of the body to sodium. For this reason, a change in the dialysate sodium concentration may not have that much of an impact on the interdialytic weight gain and blood pressures unless the sodium level is individualised. This was shown in a study conducted by Radhakrishnan et al. where they compared a set dialysate sodium concentration of 140 meq/l to an individualised dialysate sodium concentration. There was a significantly lower interdialytic weight gain and pre-dialysis systolic blood pressure in those patients who had an individualised dialysate sodium concentration in comparison to the standard dialysate sodium concentration of 140 meq/l [16].

This study showed no significant difference between the systolic or diastolic blood pressure in both dialysate sodium concentration groups. Charra et al. described the concept of ‘lag time’, whereby it takes several months for the correction of the extracellular volume overload (in our case from the high sodium) to manifest as improvement in blood pressure [18]. This study was 12 weeks long and therefore patients may not have completed this lag time.

An important finding on the study was the average interdialytic weight gain regardless of the dialysate sodium concentration used. The mean interdialytic weight gain was 2.14 kg and 2.35 kg for dialysate sodium 137 meq/l and 140 meq/l respectively. Comparing to other studies, for instance the PanThames renal audit done by Davenport et al. undergoing dialysis three times a week showed an interdialytic weight gain range of 1.7 to 2.75 kg [19]. Therefore, interdialytic weight gain in the this study is comparable to the interdialytic weight gains in the audit, despite the fact that our patient population was on twice weekly as opposed to thrice weekly dialysis. This is an interesting finding given that there are significant resource constraints in Kenya and therefore patients who are recommended to dialyse thrice weekly are dialysing twice weekly since the NHIF only covers dialysis twice a week. This raises the question as to whether twice a week dialysis is sufficient for our population considering the interdialytic weight gain is not drastically high, and in effect mortality and adverse cardiovascular outcomes may also not be so high. This also raises the question as to whether our patient population is adhering to fluid restriction and salt restriction practices more than patients in other parts of the world or whether diuretics in patients with residual renal function have an effect on this.

It is recommended that the same study be conducted using a larger sample size to assess the association between dialysate sodium concentration and interdialytic weight gain. It would also be beneficial to do a comparison study of the same outcomes of interdialytic weight gain and blood pressure control in patients on twice weekly versus thrice weekly dialysis to ascertain whether the different dialysate sodium concentrations are affected by the frequency of dialysis. A limitation of the study was that pre-dialysis sodium concentrations were not obtained and hence dialysate plasma sodium gradient was not calculated. Also, the study duration was short, and therefore as far as blood pressure is concerned, it would take time for the effects of a reduction of dialysate sodium to reduce the extracellular volume and for this to have any effect on reduction in blood pressure.

## Conclusion

In summary, our study showed there was no difference in the interdialytic weight gain between the low dialysate sodium concentrations (DNa 137 meq/l) and high dialysate sodium concentration (DNa 140 meq/l) for patients undergoing twice-weekly haemodialysis. The interdialytic weight gain in both groups was however, generally lower compared to other studies despite patients undergoing twice-weekly dialysis (as opposed to thrice-weekly). Furthermore, the blood pressures were also not different in either dialysate sodium group and this was similar to some studies. However our study did not take into account residual renal function, dietary fluid and salt intake. In addition, the duration of the study was too short to compare blood pressures amongst the two groups due to the lag time between changes in dialysate sodium and the correction of the extracellular volume. There is the possibility of clinical significance, despite no statistical significance for both interdialytic weight gain and blood pressures, since both were lower in the low DNa group compared to the high DNa group.

## Data Availability

The datasets use for this study are available from the corresponding author on reasonable request.

## Abbreviations

AKUH: Aga Khan University Hospital
CKD: Chronic Kidney Disease
DNa: Dialysate Sodium Concentration
ESRD: End-Stage Renal Disease
HIV: Human Immunodeficiency Virus
IDWG: Interdialytic Weight Gain
KDIGO: Kidney Disease Improving Global Outcomes
KDOQI: Kidney Disease Outcomes Quality Initiative
MAP: Mean Arterial Pressure
Na: Sodium
NHIF: National Health Insurance Fund
PKC: Parklands Kidney Centre
SLE: Systemic Lupus Erythematosus
